# A New Mutation in Blau Syndrome

**DOI:** 10.1155/2015/463959

**Published:** 2015-01-27

**Authors:** Cengiz Zeybek, Gokalp Basbozkurt, Davut Gul, Erkan Demirkaya, Faysal Gok

**Affiliations:** ^1^Pediatric Nephrology Department, Gulhane Military Medical Academy, Etlik, Kecioren, 06010 Ankara, Turkey; ^2^Pediatrics Department, Gulhane Military Medical Academy, Etlik, Kecioren, 06010 Ankara, Turkey; ^3^Medical Genetics Department, Gulhane Military Medical Academy, Etlik, Kecioren, 06010 Ankara, Turkey; ^4^Pediatric Rheumatology and Nephrology Department, Gulhane Military Medical Academy, Etlik, Kecioren, 06010 Ankara, Turkey

## Abstract

Blau syndrome is a rare, autosomal dominant, granulomatous autoinflammatory disease. The classic triad of the disease includes recurrent uveitis, granulomatous dermatitis, and symmetrical arthritis. Blau syndrome is related to mutations located at the 16q12.2–13 gene locus. To date, 11 NOD2 gene mutations causing Blau syndrome have been described. Here, we describe a 5-year-old male patient who presented with Blau syndrome associated with a novel sporadic gene mutation that has not been reported previously.

## 1. Background

Blau syndrome (OMIM 186580) is a rare, autosomal dominant (AD), and granulomatous autoinflammatory disease first described by Blau in 1985 [1, 2]. The classic triad of the disease includes recurrent uveitis, granulomatous dermatitis, and symmetrical arthritis; however, all symptoms may not appear concurrently [2]. Blau syndrome is associated with mutations located at the 16q12.2–13 gene locus [3]. The NOD2 gene is associated with the innate immune system [4]. To date, 11 NOD2 gene mutations causing Blau syndrome have been described. Moreover, seven NOD2 mutations that may be associated Blau syndrome have been identified [5, 6]. Here, we describe a novel sporadic gene mutation causing Blau syndrome that has not been reported previously.

## 2. Case

**Figure 1:**
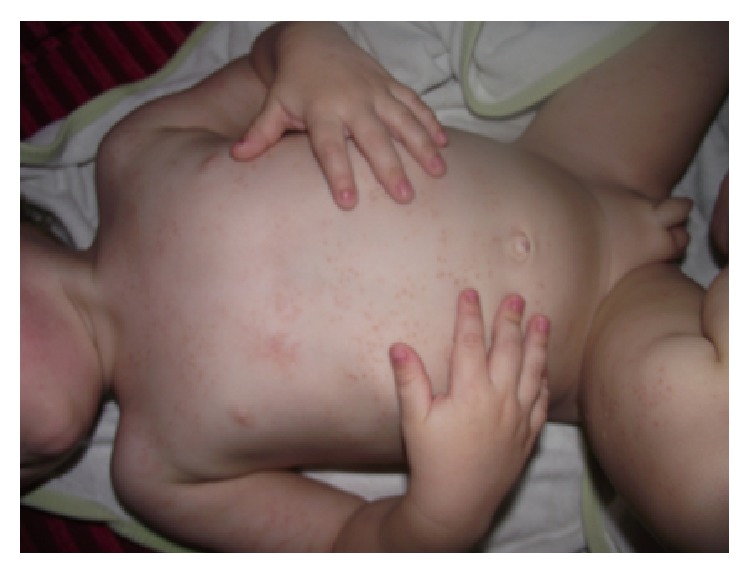
Maculopapular erythematous rash at 5 months of age.

**Figure 2:**
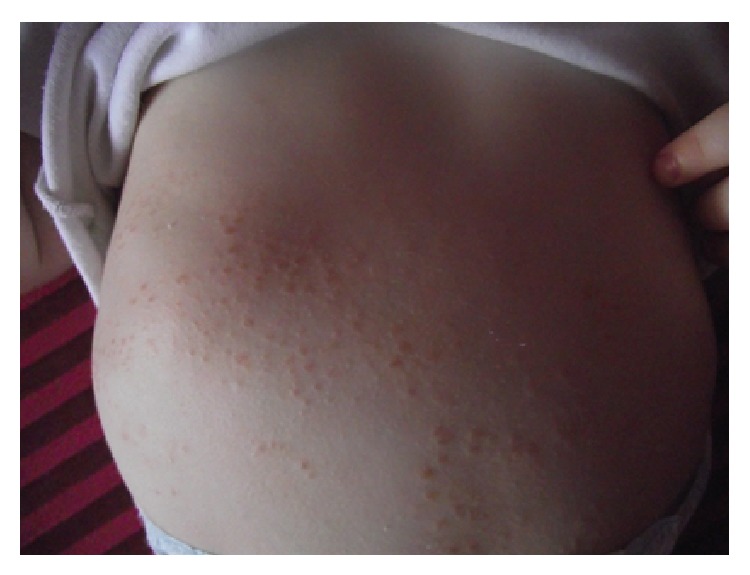
Maculopapular erythematous rash at 5 months of age.

**Figure 3:**
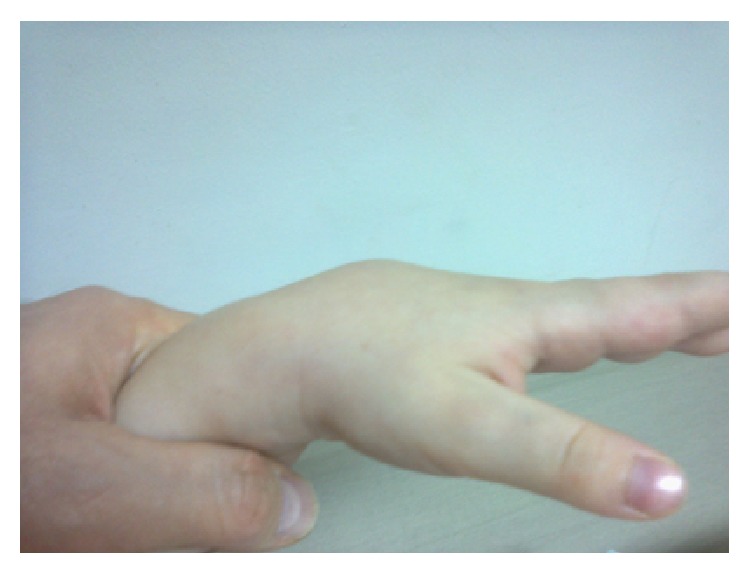
Swelling of the dorsum of the hands at 3 years of age.

A 5-year-old male was initially admitted to our clinic at 5 months of age with a maculopapular erythematous rash over his entire body (Figures 1 and 2). The patient's family history revealed no inherited familial disease. The patient's skin lesions persisted for 1 year and disappeared spontaneously. At the age of 3 years, the patient visited our unit with swelling of the dorsum of the hands (Figure 3). Physical examination revealed swelling, pain, limitation of movement, and increased warmth in both hands, but no rash. The laboratory results were as follows: hemoglobin 12.7 g/dL, white blood cell count 12600/mm^3^, platelet count 446000/mm^3^, erythrocyte sedimentation rate 15 mm/h, C-reactive protein 4.2 mg/dL (normal range, 0–0.5), rheumatoid factor 6 IU/mL (normal level, <16), and antinuclear antibody negative. Ultrasonography of the swelling, performed at another facility, revealed tenosynovial cysts. At follow-up after a 2-month period without treatment, we detected swelling of the dorsum of both feet. Four months after the first swelling appeared, the patient presented at our hospital with symmetrical arthritis of both knees. We diagnosed the patient with systemic or RF-negative polyarticular juvenile idiopathic arthritis (JIA) and administered ibuprofen, prednisolone, and subcutaneous methotrexate. Four months after treatment commenced, we detected symmetrical arthritis of both wrists and ankles. We discontinued the initial treatment and began etanercept treatment. However, the patient developed etanercept-induced fever 3 weeks after initiation of the drug treatment, and the etanercept administration was therefore ceased. During follow-up at 5 years of age, the ophthalmological examination revealed granulomatous anterior uveitis in the patient's right eye, as indicated by large precipitates in the anterior chamber and nodules in the iris. Thus, the presence of skin lesions, granulomatous ophthalmologic inflammation, and the NOD2 gene mutation ruled out the diagnosis of JIA. Taken together, the clinical and laboratory findings of our case suggested a diagnosis of Blau syndrome. Genetic studies were run to investigate the NOD2 gene mutation. The results showed only a P507S mutation; however, a novel heterozygote mutation P507S (c.1519C>T) in the fourth exon of the NOD2 gene was revealed. Analysis of the protein variant revealed that the mutation was p.Pro507Ser.* In silico* assessment (SIFT, Mutation Taster, and Polyphen) of the mutation indicated a strong association with Blau syndrome. We found no data to indicate that this mutation caused NF-kappa B hyperactivation. This heterozygous NOD2 gene mutation has not been reported previously.

Although Blau syndrome is an AD inherited disease, the parents of the patient were healthy. Blau syndrome resulting from* de novo* mutations may present sporadically [7], and we believe a* de novo* mutation caused the Blau syndrome in our case. The symptoms of the disease tend to appear before the age of 3 or 4 years [8]. The initial symptoms are primarily cutaneous and articular findings, as was the case in our patient [9–11]. Ocular findings typically appear between the ages of 7 and 12 years [1, 12]. Patients presenting with joint findings are often misdiagnosed as JIA in the absence of ocular and cutaneous findings. Granulomatous inflammation of the hand dorsum may cause tenosynovial cysts, as we observed in our case [5]. No specific cutaneous findings are associated with Blau syndrome, and most types of skin findings may occur [5]. Skin lesions may recover spontaneously after several months, as occurred in our case [5]. The histologic examination of the skin lesions revealed noncaseating granulomatous disease [5]. The eye lesions most frequently associated with Blau syndrome are recurrent anterior uveitis and panuveitis [13], which are serious and can cause blindness [5]. Steroids are the principle treatment for Blau syndrome. If steroid therapy is ineffective or causes adverse effects, biological agents including tumor necrosis factor inhibitors and interleukin-1 receptor antagonists may be used [10, 11, 14–17]. The response to these agents has been found to be variable. Thalidomide treatment has been reported to be an alternative treatment option [18]. Our patient did not achieve remission with steroid therapy; thus, he is currently receiving canakinumab therapy.

## 3. Conclusion

To date, 18 different NOD2 mutations associated with Blau syndrome have been identified. Here, we described a* de novo* mutation causing Blau syndrome that has not been reported previously.

## Abbreviations

JIA: Juvenile idiopathic arthritis
AD: Autosomal dominant.

## References

[1] Punzi L., Gava A., Galozzi P., Sfriso P. (2011). Miscellaneous non-inflammatory musculoskeletal conditions. Blau syndrome. *Best Practice & Research: Clinical Rheumatology*.

[2] Blau E. B. (1985). Familial granulomatous arthritis, iritis, and rash. *The Journal of Pediatrics*.

[3] Tromp G., Kuivaniemi H., Raphael S. (1996). Genetic linkage of familial granulomatous inflammatory arthritis, skin rash, and uveitis to chromosome 16. *The American Journal of Human Genetics*.

[4] Ogura Y., Inohara N., Benito A., Chen F. F., Yamaoka S., Núñez G. (2001). Nod2, a Nod1/Apaf-1 family member that is restricted to monocytes and activates NF-*κ*B. *The Journal of Biological Chemistry*.

[5] Sfriso P., Caso F., Tognon S., Galozzi P., Gava A., Punzi L. (2012). Blau syndrome, clinical and genetic aspects. *Autoimmunity Reviews*.

[6] Chauhan K., Michet C. (2014). A case of Blau syndrome. *Case Reports in Rheumatology*.

[7] Pillai P., Sobrin L. (2013). Blau syndrome-associated uveitis and the *NOD2* gene. *Seminars in Ophthalmology*.

[8] Rybicki B. A., Maliarik M. J., Bock C. H. (1999). The Blau syndrome gene is not a major risk factor for sarcoidosis. *Sarcoidosis Vasculitis and Diffuse Lung Disease*.

[9] Becker M. L., Rose C. D. (2005). Blau syndrome and related genetic disorders causing childhood arthritis. *Current Rheumatology Reports*.

[10] Rosé C. D., Wouters C. H., Meiorin S. (2006). Pediatric granulomatous arthritis: an international registry. *Arthritis and Rheumatism*.

[11] Aróstegui J. I., Arnal C., Merino R. (2007). *NOD2* gene-associated pediatric granulomatous arthritis: clinical diversity, novel and recurrent mutations, and evidence of clinical improvement with interleukin-1 blockade in a Spanish cohort. *Arthritis and Rheumatism*.

[12] Okafuji I., Nishikomori R., Kanazawa N. (2009). Role of the NOD2 genotype in the clinical phenotype of Blau syndrome and early-onset sarcoidosis. *Arthritis and Rheumatism*.

[13] Kurokawa T., Kikuchi T., Ohta K., Imai H., Yoshimura N. (2003). Ocular manifestations in Blau syndrome associated with a CARD15/Nod2 mutation. *Ophthalmology*.

[14] Raphael S. A., Blau E. B., Zhang W. H., Hsu S. H. (1993). Analysis of a large kindred with Blau syndrome for HLA, autoimmunity, and sarcoidosis. *The American Journal of Diseases of Children*.

[15] Simonini G., Xu Z., Caputo R. (2013). Clinical and transcriptional response to the long-acting interleukin-1 blocker canakinumab in Blau syndrome-related uveitis. *Arthritis and Rheumatism*.

[16] la Torre F., Lapadula G., Cantarini L., Lucherini O. M., Iannone F. (2014). Early-onset sarcoidosis caused by a rare CARD15/NOD2 de novo mutation and responsive to infliximab: a case report with long-term follow-up and review of the literature. *Clinical Rheumatology*.

[17] Martin T. M., Zhang Z., Kurz P. (2009). The NOD2 defect in Blau Syndrome does not result in excess interleukin-1 activity. *Arthritis and Rheumatism*.

[18] Yasui K., Yashiro M., Tsuge M. (2010). Thalidomide dramatically improves the symptoms of early-onset sarcoidosis/Blau Syndrome: its possible action and mechanism. *Arthritis and Rheumatism*.

